# The association between the workload of general practitioners and patient experiences with care: results of a cross-sectional study in 33 countries

**DOI:** 10.1186/s12960-020-00520-9

**Published:** 2020-10-16

**Authors:** Willemijn L. A. Schäfer, Michael J. van den Berg, Peter P. Groenewegen

**Affiliations:** 1 Department of Surgery, Feinberg School of Medicine, Northwestern University, Chicago, IL 60611 USA; 2 Department of Public Health, Academic Medical Center, Amsterdam, the Netherlands; 3 NIVEL, the Netherlands Institute for Health Services Research, Utrecht, The Netherlands; 4 Department of Human Geography, Utrecht University, Utrecht, The Netherlands; 5 Department of Sociology, Utrecht University, Utrecht, The Netherlands

**Keywords:** General practice, Workload, Job satisfaction, Patient experiences, International comparison

## Abstract

**Background:**

The workload of general practitioners (GPs) and dissatisfaction with work have been increasing in various Western countries over the past decades. In this study, we evaluate the relation between the workload of GPs and patients’ experiences with care.

**Methods:**

We collected data through a cross-sectional survey among 7031 GPs and 67,873 patients in 33 countries. Dependent variables are the patient experiences on doctor-patient communication, accessibility, continuity, and comprehensiveness of care. Independent variables concern the workload measured as the GP-reported work hours per week, average consultation times, job satisfaction (an indicator of subjective workload), and the difference between the workload measures of every GP and the average in their own country. Finally, we evaluated interaction effects between workload measures and what patients find important in a country and the presence of a patient-list system. Relationships were determined through multilevel regression models.

**Results:**

Patients of GPs who are happier with their work were found to experience better communication, continuity, access, and comprehensiveness. When GPs are more satisfied compared to others in their country, patients also experience better quality. When GPs work more hours per week, patients also experience better quality of care, but not in the area of accessibility. A longer consultation time, also when compared to the national average, is only related to more comprehensive care. There are no differences in the relationships between countries with and without a patient list system and in countries where patients find the different quality aspects more important.

**Conclusions:**

Patients experience better care when their GP has more work hours, longer consultation times, and especially, a higher job satisfaction.

## Background

Ageing of populations, changes in lifestyle, and the associated increase of multi-morbidity have led to more and more complex health problems [1–4]. Primary care is increasingly important to address the needs of patients and populations, as is acknowledged both by the World Health Organization [5] and the European Union [6].

The increase in patients with complex health problems changes the work of general practitioners (GPs) and increases their workload. This is for example visible in the increase of the consultation length in the United Kingdom, the United States of America [7], and the Netherlands [8]. Additionally, job satisfaction, a subjective aspect of workload [9], is low and decreasing among primary care physicians in various countries [10–13]. An increased workload has important consequences for patients. When GPs are under time pressure, this may negatively affect the time they have to discuss patients’ health problems. This can be a particular problem for patients with complex health problems, including chronic diseases or comorbidities, who may need additional time to have their health issues addressed [14]. Whereas many previous studies focused on the determinants of the workload of GPs (see for example [15–23]), studies on the consequences of high workload in GPs for patient experiences are sparse. Previous studies showed that extended consultation length is related to increased patients’ ability to cope with their illness and life in general [24], and more positive experiences with the care provided by GPs [25]. GPs, who more frequently experienced a lack of time, did not differ from other GPs on most communication aspects, except for patient-centredness [26]. More work hours are found to be associated with better perceived availability and accessibility in one study [25] but not in another [27]. Additionally, more work hours are related to more positive patient evaluations on the doctor-patient relationship and medical care and information, but not on accessibility [27].

The current study adds to this literature through studying patients’ experiences in four key domains of primary care: accessibility, continuity, and comprehensiveness of care and doctor-patient communication. We study the associations between experiences and three aspects of GP workload: the number of work hours, consultation length, and job satisfaction. The main research question is as follows: What is the relationship between the workload of GPs and patients’ experiences of care? Data is used on a large sample of patients and the GPs they consulted in 33 countries in Europe and outside, with a high variation in the workload and patient experiences [28, 29]. The use of data on a large number of countries allows us to take into account country characteristics in the data analyses.

Longer consultations and a higher job satisfaction are expected to be associated with more positive patient experiences. A higher number of work hours are expected to be associated with more negative experiences, but this can be mitigated by a higher job satisfaction; GPs with more working hours but at the same time a high job satisfaction would be less likely to yield negative patient experiences. The average number of working hours and average consultation time differ between countries. Consequently, the same values may in one country be in the lower end of distribution, but in another country in the higher end. It is therefore important also to look at the deviation from the average within countries. When GPs have shorter work hours, longer consultations, and a higher job satisfaction compared to other GPs in their country, the relation with more positive patient experiences is expected to be stronger. Furthermore, if patients in a country find the experience domain (access, comprehensiveness, continuity, communication), more important, the association with work hours, consultations, and a higher job satisfaction is expected to be more stronger. Finally, we expect that in countries without a patient list system, patients only choose to visit a family physician over another specialist because the family physician better meets their expectations. Therefore, we would expect that the association with the workload variables is stronger in countries where patients are enrolled with a specific GP compared to countries without a list system.

## Methods

### Data collection

Data used in this article were retrieved from the QUALICOPC (Quality and Costs of Primary Care in Europe) study. Within this European Commission-funded study, data were collected among GPs and their patients in 31 European countries (EU 27—except France—, Iceland, Norway, the Republic of Macedonia, Turkey, and Switzerland) between 2011 and 2013. In addition, we used the QUALICOPC data from two non-European countries with health systems comparable to European systems (Canada and New Zealand). In each country, a sample of GPs completed a questionnaire. For most countries, the number of respondents was around 220 GPs. For smaller countries (Cyprus, Iceland, Luxembourg, and Malta), this was around 75 GPs. In most countries, a random sample of GPs was invited to participate. In countries where no national sampling framework was available, alternatives were sought as close as possible to a random sample. Per practice, only one GP participated [30].

In each practice, ten consecutive patients who visited the GP who participated in the study were invited by a fieldworker to complete a questionnaire; nine patients completed a questionnaire about their experiences with the consultation they just had, and one patient completed a questionnaire about what s/he considers important in the care of GPs. The questions are derived from validated questionnaires. Details of the study design and the development of the questionnaires have been published elsewhere [31, 32]. Ethical review was conducted in accordance with the legal requirements in each country [33].

### The dependent variables: patient experiences

The questionnaire for patients included questions about the perceived accessibility (five questions), continuity (three questions), and comprehensiveness of care (two questions) and doctor-patient communication (three questions). The patients answered the questions with ‘yes’ or ‘no’. Patients were asked, for example, whether the doctor made eye contact during the consultation as an indicator for communication and whether the doctor had their personal medical records at hand as an indicator for continuity of care. The questions were combined in a composite scale score for each of the four areas using multilevel, ecometric analysis [34, 35] in which the items are nested in patients, nested in the GP practices, and nested in countries. The resulting scale values (which vary between 0 and 1) are multiplied by 100 for easily interpretable regression coefficients.

### Independent variables

Within this study, objective workload is defined as ‘the amount of time that certain activities consume or the frequency that certain activities take place’ [9]. To measure workload, we have selected two indicators that fall within this definition: the regular working hours and average consultation length. GPs reported estimates of the average number of *work hours per week*, including the regular hours, but excluding evening, night, and weekend shifts. The survey does not distinguish between full-time or part-time working status. To measure *consultation length*, the GPs were asked to estimate how long a regular patient consultation in their office usually takes (in minutes).

*Job satisfaction*, representing subjective workload of GPs, was measured using a scale score on six items derived from the European Task Profile Study [36]. The items are:

- I feel that some parts of my work do not really make sense.
- My work still interests me as much as it ever did.
- My work is overloaded with unnecessary administrative detail.
- I have too much stress in my current job.
- Being a general practitioner is a well-respected job.
- In my work, there is a good balance between effort and reward.

For each item, the GPs indicated to what extent they agreed on a scale of 1 (strongly disagree) to 4 (strongly agree). The items were coded so that a higher score indicates higher satisfaction. We constructed a scale using ecometric analysis, in which items are nested in GPs and nested in countries.

We calculated the deviation from the national average, by subtracting the national average from the individual values of each GP. In addition, we calculated the average importance that patients attach to the four aspects of the quality of GP care. Country-level scales for each domain were constructed using latent multilevel regression analyses. In the models, we adjusted for the age, gender, level of household income, ethnicity, and level of education of the patients [37].

### Confounders

We included the GP characteristics, age (centred) and sex. At the patient level, we included several sociodemographic variables: age (centred), sex, perceived health (poor, fair, good, very good), the presence of one or more chronic diseases (yes or no), ethnicity (first-generation, second-generation immigrant, native), education (low, medium, high), and income (below average, average, above average). Sociodemographic variables are related to patient experiences [38, 39].

### Statistical analysis

Within this study, we focus on the independent association between the three workload variables and the experiences of patient. To determine the associations, we have performed multi-level linear regression with three levels: country, GP, and patient. Consequently, variation in patient experiences is at three levels, and we have calculated the intraclass correlations (ICC) at GP and country level, as the percentage of total variation residing at that level. The analyses were done for each of the four patient experience outcome variables using a stepwise approach with 11 steps. In the first step, an empty model was estimated to show how the patient experiences are clustered on different levels. In the second model, we added the confounders. In the third model, we added the indicators for workload. In the fourth model we added the interaction term for satisfaction and work hours. In the fifth model, we analysed the individual deviations on the workload variables from the averages instead of the absolute values of the workload variables (confounders in the model). In models 6 to 8, we returned to model 4 and added one by one the interactions between the importance that the patients in each country attached to the accessibility, communication, comprehensiveness, and continuity of care and the three workload indicators. In models 9 to 11, we analysed the interactions between the presence of a list system and the three workload indicators (added to model 4). The complete 11-step model results are in Additional file 1: Appendix Tables 2–5. We used the software programme Stata (version 14.2).

## Results

The total number of participating GPs amounts to 7031. In total, 60,741 patients completed the questionnaire on experiences, and 7132 patients about what they find important.

### Variation in patient experiences

**Fig. 1:**
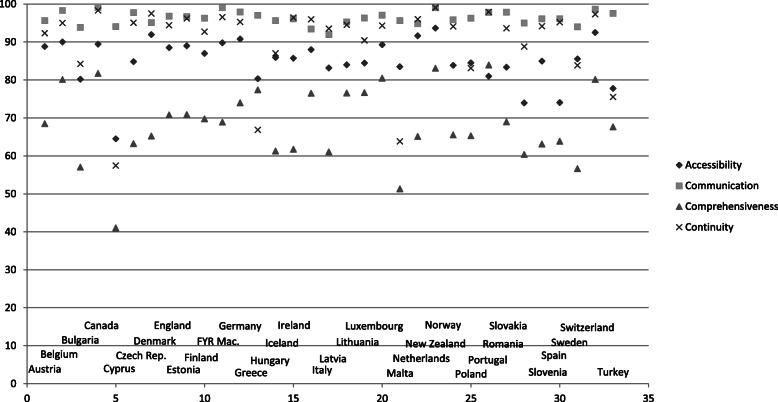
Average values of patient experiences scale values by country and domain

The average values for the patient experiences on accessibility range from 64.5 in Cyprus to 93.7 in New Zealand, for communication from 91.9 in Italy to 99.1 in Canada, for comprehensiveness from 41.0 in Cyprus to 83.9 in Portugal, and for continuity from 57.4 in Cyprus to 99.0 in New Zealand (see Fig. 1 and Additional file 1: Appendix Table 1). The experience of the physician-patient communication mostly varies between patients (more than half of the variation), but there is also substantial variation between GPs (36% of the variation) and between countries (12% of the variation). The variation in the perceived continuity of care is more than one third at the patient level, more than one third at the country level, and about a quarter at the level of GPs. The distribution of the variation in experience of the accessibility and comprehensiveness of care is completely different: there is hardly any variation between patients (respectively 4 and 2%), but especially among GPs (respectively 55 and 53%) and countries (respectively 41 and 45%) (see Additional file 1: Appendix Tables 2–5).

### Workload and job satisfaction of GPs

The average number of hours worked varies from 33.5 h in Italy to 51.1 h per week in Belgium. The average consultation time varies from about 8 min in Hungary to 24 min in Finland and Sweden (see Table 1). GPs in Denmark are most satisfied with their job, whereas the Spanish and Hungarian GPs reported on average the lowest job satisfaction.

**Table 1:** Average number of work hours, duration of consultations, and job satisfaction score of GPs in 33 countries (standard deviation)

	GPs in QUALICOPC database (*n*)	Work hours per week	Duration of consultations in minutes	Job satisfaction score
Austria	184	43.7 (12.5)	11.7 (12.4)	2.44 (.30)
Belgium	408	51.1 (13.1)	17.7 (5.9)	2.59 (.27)
Bulgaria	223	39.2 (13.5)	17.6 (6.0)	2.44 (.28)
Canada	792	40.4 (11.8)	14.8 (9.2)	2.77 (.30)
Cyprus	71	37.5 (5.8)	18.3 (9.0)	2.81 (.21)
Czech Republic	291	36.2 (8.9)	10.9 (4.8)	2.49 (.23)
Denmark	212	40.9 (7.3)	14.3 (2.1)	2.97 (.27)
England	171	40.1 (10.5)	11.2 (2.1)	2.49 (.32)
Estonia	137	37.8 (10.0)	16.4 (3.8)	2.27 (.23)
Finland	288	35.8 (7.6)	23.8 (6.3)	2.59 (.30)
FYR Macedonia	143	40.8 (3.1)	13.4 (4.8)	2.35 (.26)
Germany	238	49.6 (9.7)	10.6 (4.0)	2.45 (.28)
Greece	220	38.2 (11.2)	14.7 (6.3)	2.62 (.28)
Hungary	222	37.7 (8.6)	8.2 (5.5)	2.17 (.31)
Iceland	80	39.8 (7.0)	19.2 (2.7)	2.50 (.28)
Ireland	169	41.2 (10.4)	12.8 (3.3)	2.60 (.28)
Italy	218	33.5 (12.9)	13.4 (4.0)	2.37 (.30)
Latvia	218	38.9 (9.8)	17.5 (6.6)	2.36 (.23)
Lithuania	225	35.2 (9.3)	15.9 (4.2)	2.27 (.27)
Luxembourg	78	45.6 (11.9)	17.6 (5.0)	2.71 (.23)
Malta	70	46.3 (13.2)	13.0 (4.2)	2.47 (.35)
Netherlands	238	43.0 (10.0)	11.1 (1.7)	2.63 (.25)
New Zealand	168	36.8 (10.5)	14.9 (3.9)	2.68 (.31)
Norway	198	36.1 (10.1)	18.6 (2.8)	2.75 (.27)
Poland	220	38.4 (7.6)	13.7 (5.1)	2.41 (.27)
Portugal	216	40.2 (4.5)	18.1 (6.6)	2.41 (.25)
Romania	220	35.8 (8.0)	16.6 (5.1)	2.38 (.28)
Slovakia	220	37.4 (8.7)	8.9 (3.5)	2.23 (.31)
Slovenia	207	37.4 (6.6)	9.6 (3.1)	2.29 (.27)
Spain	428	35.8 (4.3)	8.5 (4.5)	2.15 (.28)
Sweden	97	34.4 (9.7)	23.9 (5.5)	2.73 (.30)
Switzerland	199	46.5 (11.6)	19.5 (5.8)	2.70 (.28)
Turkey	299	40.8 (4.1)	9.3 (5.6)	2.30 (.28)

### The relationship between patient experiences and GP workload

Table 2 provides a summary of the analyses on the relation between workload and patient experiences. Additional file 1: Appendix Tables 2–5 provide the detailed outcomes of all models, including numbers of observations for all levels, all regression coefficients, variances for all levels, and intraclass correlations. The patients of GPs who *work more* hours per week experience better communication, continuity, and comprehensiveness of care. For GPs with a longer working week compared to the average in their country, patient experiences on continuity and comprehensiveness are also more positive. The relation between patient perceived continuity and work hours is also stronger when the GPs are more satisfied. A longer *consultation time* is only related to better comprehensiveness, and this is also true for GPs with longer consultations than the national average. Patients of GPs who are more *satisfied with their work* have more positive experiences with communication, accessibility, continuity, and comprehensiveness of care. These relationships are also found for physicians with a higher job satisfaction compared to others in their country*.* After adding all workload variables to the model, we found small reductions in the variances of up to 2.8% for accessibility at the country level (Additional file 1: Appendix Tables 2–5, percentages of change not indicated in table). We did not find differences in the relation between workload and patient experiences between countries with and without a patient list system and in countries where patients find communication, continuity, accessibility, and comprehensiveness more important.

**Table 2:** Summary multilevel regression coefficients (with 95% C.I.) of aspects of workload and job satisfaction of GPs and patient experiences (for complete models, see Additional file 1: Appendix Tables 2–5)

	Communication	Continuity	Accessibility	Comprehensiveness
**Model 3: Workload variables (main effects)**				
**Work hours per week (average)**	0.01 (0.00–0.02)*	0.05 (0.03–0.08)**	0.02 (− 0.00 to 0.04)	0.04 (0.01–0.07)**
**Consultation time (minutes)**	− 0.002 (− 0.02 to 0.01)	0.02 (− 0.03 to 0.06)	− 0.01 (− 0.04 to 0.03)	0.11 (0.06–0.16)**
**Job satisfaction (scales 1–4)**	0.55 (0.27–0.82)**	1.44 (0.63–2.26)**	1.19 (0.58–1.80)**	1.76 (0.81–2.72)**
**Model 4: Interaction between job satisfaction and work hours**				
**Work hours × job satisfaction**	− 0.02 (− 0.04 to 0.01)	0.04 (− 0.03 to 0.10)	− 0.02 (− 0.07 to 0.03)	0.01 (− 0.06 to 0.09)
**Model 5: Deviation from national averages**				
**Deviation national average work hours**	0.01 (− 0.00 to 0.02)	0.05 (0.03–0.08)**	0.02 (− 0.00 to 0.03)	0.04 (0.01–0.07)**
**Deviation national average cons. time**	− 0.002 (− 0.02 to 0.01)	0.02 (− 0.03 to 0.06)	− 0.01 (− 0.04 to 0.03)	0.11 (0.06–0.16)**
**Deviation national average job satisfaction**	0.55 (0.27–0.82)**	1.45 (0.64–2.27)**	1.17 (0.56–1.78)**	1.76 (0.80–2.71)**
**Models 6–8: Interactions between patient values and workload**				
**Values × work hours**	0.04 (− 0.03 to 0.11)	− 0.06 (− 0.21 to 0.10)	0.06 (− 0.13 to 0.25)	− 0.06 (− 0.17 to 0.05)
**Values × consultation time**	0.12 (− 0.00 to 0.24)	0.19 (− 0.07 to 0.45)	0.07 (− 0.04 to 0.17)	0.09 (− 0.13 to 0.31)
**Values × job satisfaction**	0.12 (− 2.15 to 2.39)	− 0.45 (− 5.41 to 4.51)	2.67 (− 0.73 to 6.07)	− 2.22 (− 6.21 to 1.77)
**Models 9–11: Interactions between patient list system and workload**				
**Patient list system × work hours**	− 0.002 (− 0.02 to 0.02)	− 0.01(− 0.06 to 0.05)	0.004 (− 0.04 to 0.04)	0.01 (− 0.05 to 0.07)
**Patient list system × consultation time**	− 0.01 (− 0.04 to 0.03)	− 0.09 (− 0.19 to 0.01)	− 0.01 (− 0.08 to 0.06)	− 0.10 (− 0.21 to 0.01)
**Patient list system × job satisfaction**	0.16 (− 0.50 to 0.82)	− 1.93 (− 3.86 to 0.001)	0.51 (− 0.92 to 0.95)	− 1.46 (− 3.70 to 0.78)

*p **< 0.05**

**p **< 0.01**

## Discussion

The shortest summary of our findings is that there is a positive association between GP job satisfaction and patient-reported experiences of care. This finding concerns all four measured areas of patient experiences. In addition, we found positive associations between the average work hours and the patient perceived communication, continuity, and comprehensiveness of care. It is likely that GPs, who work more hours, are more often available for consultations and therefore know their patients and their medical backgrounds better (continuity), take more time to ask about various aspects of the patients’ health (comprehensiveness), and pay more attention to their communication.

### Main findings compared to other studies

A study in the United Kingdom [40] did not find an association between consultation length and patient experiences in the areas of communication, trust and confidence, and overall satisfaction. We only found an association of consultation length with experienced comprehensiveness of care, but also not with communication.

Previous studies found that more work hours are related to a broader availability of people’s ‘own’ GP [25, 27]. This is important against the background of the increasing numbers of female GPs who tend to work less hours [41]; in general, part-time working is on the increase among GPs. We did find that patients of GPs with more work hours experience more comprehensiveness, measured as their doctor discussing multiple problems, including personal problems. One study found no relationship between the workload of GPs and their awareness of psychological problems [26], but another study found indications that longer consultations led to a better diagnosis of psychological problems [42].

In addition to the experiences of patients, it is also important to consider the associations between workload and clinical quality of care. Other studies found that GPs under time pressure provided reduced quality of care during physical examination [43] and a less thorough clinical examination and gave less advice on lifestyle [44]. A positive relation between the workload of GPs and cardiovascular prevention was found [45]. No relation was found between GP workload and adherence to guidelines [46].

### Limitations and strengths

This study has some limitations. Firstly, the data on workload are based on self-reports from GPs and not on actual time measurements. Hence, the actual number of hours worked or the consultation length might differ from the reported figures. The number of work hours could be underestimated by GPs, as was observed in the Netherlands [47]. However, we expect that this does not affect the comparisons between countries. Secondly, the recruitment and participation of GPs in the study differs between countries, even though we attempted to implement the study as uniform as possible. GPs who feel overloaded may have decided not to participate. Thirdly, only patients who visited their GP completed the patient questionnaire. Patients without access to their GP were not reached. Another potential limitation is that the patient experience scales were developed from previously validated survey questions and scales. However, some questions were adapted and not validated in their current form. Finally, the associations presented in this analysis are based on cross-sectional data. Therefore, causal interpretation is not warranted.

The study has several strengths. We use comparable data on many countries with large numbers of GPs and patients. The response groups are largely representative for the GP population by gender and average age [30]. A limitation, but at the same time a strength, is that this study only evaluated the experiences of patients with their GPs. This is a strength because it makes the direct link between GP questionnaires and the questionnaires of their patient possible. A limitation is that in modern primary care practices, care is also provided by other professionals, such as practice nurses. Future research on the relationship between workload and patient experiences should therefore also look at the experiences of patients with other staff in primary care in relation to their workload.

### Implications for policy, practice, or research

The results of this study show that job satisfaction does not only relate to physician burnout and clinical quality of care, but also to patient experiences. GP job satisfaction varies between countries, with Spanish, Hungarian, Slovakian, Estonian, and Latvian doctors being least satisfied. Potential determinants of the job satisfaction of GPs include the electronic medical records system [23], the way out-of-hours service delivery is organized [48–51], the payment system [52], the practice location [53], and the workload of GPs. Results of studies on the workload and the job satisfaction of GPs are, however, ambiguous. A number of studies found no relation between workload and satisfaction [54, 55] or subjective workload [23]. On the other hand, another study found that higher workload resulted in lower satisfaction with work [44]. A longer consultation is associated with less stress from the GP [24, 56].

In addition, the work hours are associated with the experiences of the patients. Countries with the lowest average work hours include Italy, Sweden, Lithuania, Romania, and Finland. In further studies, the impact of continuous availability of the same GP needs to be studied. This may be particularly relevant for patients with complex diseases such as cancer or diabetes. Finally, longer consultation times, more work hours, and job satisfaction were all related to the patient-perceived comprehensiveness. The option to discuss multiple problems during consultations is especially important for patients with multimorbidity. Globally, there are large differences in consultation times [7].

### Conclusion

The subjective and objective workload of GPs is related to the experiences of patients. We found that GPs provide a better patient experience when they work longer hours, have longer consultation times, and have high levels of job satisfaction.

## Supplementary information

**Additional file 1:.** Appendix Tables.

## Data Availability

The datasets used and/or analysed during the current study are available from the corresponding author on reasonable request.

## Abbreviations

FYR Macedonia: Former Yugoslav Republic of Macedonia
GP(s): General practitioner(s)
ICC: Intraclass correlation
QUALICOPC: Quality and Costs of Primary Care in Europe
