# Supplementary material for: The association between the workload of general practitioners and patient experiences with care: results of a cross-sectional study in 33 countries
Source: Hum Resour Health. 2020 Oct 16;18:76. doi: 10.1186/s12960-020-00520-9 (PMC7565810; doi:10.1186/s12960-020-00520-9)
Supplement: Supplementary file 1 — Additional file 1:. Appendix Tables. [file 12960_2020_520_MOESM1_ESM.docx]

Appendix 1: Average values of patient experiences scale values by country by domain (Range 1-100)

| **Country** | **Accessibility** | **Communication** | **Comprehensiveness** | **Continuity** |
| --- | --- | --- | --- | --- |
| Austria | 88.8 | 95.6 | 68.5 | 92.3 |
| Belgium | 90.0 | 98.3 | 80.1 | 95.0 |
| Bulgaria | 80.2 | 93.8 | 57.0 | 84.2 |
| Canada | 89.4 | 99.1 | 81.7 | 98.3 |
| Cyprus | 64.5 | 94.1 | 41.0 | 57.4 |
| Czech Republic | 84.8 | 97.8 | 63.2 | 95.1 |
| Denmark | 92.0 | 95.1 | 65.2 | 97.5 |
| England | 88.5 | 96.8 | 70.8 | 94.4 |
| Estonia | 89.0 | 96.7 | 70.9 | 96.2 |
| Finland | 87.0 | 96.3 | 69.7 | 92.7 |
| FYR Macedonia | 89.8 | 99.0 | 68.9 | 96.5 |
| Germany | 90.8 | 97.9 | 74.0 | 95.3 |
| Greece | 80.3 | 97.0 | 77.4 | 66.8 |
| Hungary | 85.9 | 95.6 | 61.3 | 87.0 |
| Iceland | 85.7 | 96.1 | 61.8 | 96.4 |
| Ireland | 88.0 | 93.4 | 76.5 | 96.0 |
| Italy | 83.2 | 91.9 | 61.0 | 93.5 |
| Latvia | 84.0 | 95.3 | 76.6 | 94.5 |
| Lithuania | 84.4 | 96.3 | 76.7 | 90.4 |
| Luxembourg | 89.3 | 97.1 | 80.5 | 94.3 |
| Malta | 83.5 | 95.6 | 51.3 | 63.8 |
| Netherlands | 91.6 | 94.8 | 65.1 | 96.0 |
| New Zealand | 93.7 | 99.1 | 83.1 | 99.0 |
| Norway | 83.8 | 95.9 | 65.5 | 94.1 |
| Poland | 84.5 | 96.3 | 65.3 | 83.1 |
| Portugal | 81.0 | 97.8 | 83.9 | 97.9 |
| Romania | 83.4 | 97.8 | 68.9 | 93.6 |
| Slovakia | 74.0 | 95.0 | 60.4 | 88.8 |
| Slovenia | 85.0 | 96.1 | 63.1 | 94.2 |
| Spain | 74.0 | 96.1 | 63.9 | 95.2 |
| Sweden | 85.5 | 94.0 | 56.6 | 83.9 |
| Switzerland | 92.5 | 98.6 | 80.1 | 97.3 |
| Turkey | 77.8 | 97.5 | 67.6 | 75.5 |

Appendix 2: Results linear multilevel analyses patient experiences with ***communication*** (regression coefficients with p-values)

| N_i_=33  N_j_= 6,512  N_k_= 56,794 | Model 1 | Model 2 | Model 3 | Model 4 | Model 5 | Model 6 |
| --- | --- | --- | --- | --- | --- | --- |
| Cons | 96.28 (0.00) | 96.23 (0.00) | 94.56 (0.00) | 92.87 (0.00) | 96.21 (0.00) | 86.42 (0.00) |
| **Patient characteristics** |  |  |  |  |  |  |
| Age (centered) |  | -.004 (0.001) | -.004 (0.001) | -.004 (0.001) | -.004 (0.001) | -.004 (0.001) |
| Gender (ref=Man) |  | .037 (0.260) | .037 (0.256) | .037 (0.257) | .037 (0.256) | .037 (0.255) |
| Perceived health (ref=good)  - Poor  - Fair  - Very good |  | -.201 (0.002)  -.073 (0.069)  -.033 (0.505) | -.201 (0.002)  -.073 (0.070)  -.033 (0.509) | -.201 (0.002)  -.073 (0.070)  -.033 (0.508) | -.201 (0.002)  -.074 (0.069)  -.033 (0.510) | -.200 (0.002)  -.073 (0.070)  -.033 (0.504) |
| Chronic disease(s) |  | .008 (0.843) | .008 (0.834) | .008 (0.831) | .008 (0.834) | .008 (0.842) |
| Ethnicity(ref=non-immigrant)  - Second generation  - First generation |  | -.100 (0.220)  -.327 (0.000) | -.100 (0.223)  -.328 (0.000) | -.100 (0.221)  -.328 (0.000) | -.100 (0.224)  -.327 (0.000) | -.100 (0.221)  -.328 (0.000) |
| Education (ref=in between)  - lowest qualifications  - Highest qualifications |  | -.095 (0.028)  .180 (0.000) | -.095 (0.028)  .180 (0.000) | -.095 (0.028)  .180 (0.000) | -.095 (0.028)  .180 (0.000) | -.095 (0.027)  .181 (0.000) |
| Hh. income (ref=average)  - below average  - above average |  | -.064 (0.097)  .044 (0.398) | -.063 (0.104)  .045 (0.396) | -.063 (0.102)  .045 (0.396) | -.063 (0.103)  .045 (0.396) | -.063 (0.104)  .044 (0.402) |
| **GP characteristics** |  |  |  |  |  |  |
| Gender (ref=Man) |  | .297 (0.000) | .330 (0.000) | .330 (0.000) | .327 (0.000) | .332 (0.402) |
| Age (centered) |  | -.0002 (0.626) | -.0002 (0.589) | -.0003 (0.584) | -.0003 (0.000) | -.0003 (0.613) |
| **Workload** |  |  |  |  |  |  |
| Working hours |  |  | .008 (0.045) | .0509 (0.071) |  | .008 (0.042) |
| Consultation duration |  |  | -.002 (0.752) | -.003 (0.723) |  | -.429 (0.053) |
| Job satisfaction |  |  | .545 (0.000) | 1.217 (0.008) |  | .545 (0.000) |
| **Deviations** |  |  |  |  |  |  |
| Hours deviation from national average |  |  |  |  | .008 (0.058) |  |
| Minutes deviation from national average |  |  |  |  | .547 (0.768) |  |
| Satisfaction deviation from national average |  |  |  |  | .547 (0.000) |  |
| **Interactions** |  |  |  |  |  |  |
| Wrk hrs X satisfaction |  |  |  | -.017 (0.127) |  |  |
| Values communication (main effect) |  |  |  |  |  | 2.33 (0.359) |
| Communication X minutes |  |  |  |  |  | .121 (0.055) |
| **Variance** |  |  |  |  |  |  |
| Country level | 2.95 (.742) | 2.801 (.704) | 2.756 (.694) | 2.755 (.694) | 2.798 (.704) | 2.523 (.638) |
| Practice level | 8.95 (.185) | 8.311 (.174) | 8.284 (.173) | 8.280 (.173) | 8.284 (.173) | 8.278 (.173) |
| Patient level | 13.165 (.083) | 11.873 (.077) | 11.873 (.077) | 11.873 (.077) | 11.873 (.077) | 11.873 (.077) |
| **ICCs** |  |  |  |  |  |  |
| ICC country | 11.8% | 12.2% | 12.0% | 12.0% | 12.2% | 11.1% |
| ICC GPs | 35.7% | 36.2% | 36.2% | 36.1% | 36.1% | 36.5% |

Appendix 2 (continued)

| N_i_=33  N_j_= 6,731  N_k_= 58,680 | Model 7 | Model 8 | Model 9 | Model 10 | Model 11 |
| --- | --- | --- | --- | --- | --- |
| Cons | 85.622 (0.000) | 81.318 (0.000) | 94.361 (0.000) | 94.361 (0.000) | 94.735 (0.000) |
| **Patient characteristics** |  |  |  |  |  |
| Age (centered) | -.004 (0.001) | -.004 (0.001) | -.004 (0.001) | -.004 (0.001) | -.004 (0.001) |
| Gender (ref=Man) | .037 (0.256) | .037 (0.255) | .047 (0.182) | .047 (0.181) | .047 (0.181) |
| Perceived health (ref=good)  - Poor  - Fair  - Very good | -.200 (0.002)  -.073 (0.071)  -.033 (0.511) | -.200 (0.002)  -.073 (0.070)  -.033 (0.504) | -.199 (0.004)  -.075 (0.086)  -.038 (0.491) | -.199 (0.004)  -.075 (0.086)  -.038 (0.492) | -.199 (0.004)  -.075 (0.085)  -.038 (0.492) |
| Chronic disease(s) | .008 (0.835) | .008 (0.842) | .009 (0.835) | .009 (0.832) | .009 (0.830) |
| Ethnicity(ref=non-immigrant)  - Second generation  - First generation | -.100 (0.222)  -.328 (0.000) | -.100 (0.221)  -.328 (0.000) | -.107 (0.260)  -.389 (0.000) | -.107 (0.261)  -.388 (0.000) | -.107 (0.261)  -.388 (0.000) |
| Education (ref=in between)  - lowest qualifications  - Highest qualifications | -.095 (0.028)  .181 (0.000) | -.095 (0.028)  .181 (0.000) | -.088 (0.053)  .202 (0.000) | -.089 (0.052)  .202 (0.000) | -.089 (0.052)  .202 (0.000) |
| Hh. income (ref=average)  - below average  - above average | -.063 (0.105)  .045 (0.396) | -.063 (0.105)  .044 (0.397) | -.066 (0.110)  .051 (0.385) | -.066 (0.111)  .051 (0.384) | -.066 (0.111)  .051 (0.385) |
| **GP characteristics** |  |  |  |  |  |
| Gender (ref=Man) | .335 (0.000) | .331 (0.000) | .359 (0.000) | .361 (0.000) | .360 (0.000) |
| Age (centered) | -.0003 (0.568) | -.0003 (0.589) | -.0002 (0.699) | -.0002 (0.705) | -.0002 (0.706) |
| **Workload** |  |  |  |  |  |
| Working hours | -.128 (0.290) | .008 (0.046) | .009 (0.042) | .011 (0.126) | .009 (0.046) |
| Consultation duration | -.002 (0.777) | -.002 (0.748) | .001 (0.929) | -.003 (0.701) | -.003 (0.700) |
| Job satisfaction | .541 (0.000) | .132 (0.974) | .575 (0.000) | .574 (0.000) | .461 (0.101) |
| **Interactions** |  |  |  |  |  |
| Values communication (main effect) | 2.548 (0.351) | 3.782 (0.306) |  |  |  |
| values X hours | .039 (0.260) |  |  |  |  |
| Values x satisfaction |  | .117 (0.920) |  |  |  |
| List system (main effect) |  |  | -.008 (0.990) | .001 (0.999) | -.5272247 (0.616) |
| List X minutes |  |  | -.007 (0.666) |  |  |
| List X hours |  |  |  | -.003 (0.747) |  |
| List X satisfaction |  |  |  |  | .160 (0.634) |
| **Variance** |  |  |  |  |  |
| Country level (s.e.) | 2.521 (.637) | 2.521 (.637) | 2.628 (.675) | 2.620 (.673) | 2.626 (.674) |
| Practice level (s.e.) | 8.282 (.173) | 8.284 (.173) | 8.977 (.196) | 8.977 (.196) | 8.977 (.196) |
| Patient level (s.e.) | 11.873 (.077) | 11.873 (.077) | 12.819 (.087) | 12.819 (.087) | 12.819 (.087) |
| **ICCs** |  |  |  |  |  |
| ICC country | 11.1% | 11.1% | 10.8% | 10.7% | 10.8% |
| ICC GPs | 36.5% | 36.5% | 36.8% | 36.8% | 36.8% |

Appendix 3: Results linear multilevel analyses patient experiences with ***continuity*** (regression coefficients with p-values)

| N_i_=33  N_j_= 6,512  N_k_= 56,996 | Model 1 | Model 2 | Model 3 | Model 4 | Model 5 | Model 6 |
| --- | --- | --- | --- | --- | --- | --- |
| Cons | 90.128 (0.000) | 89.164 (0.000) | 83.181 (0.000) | 87.088 (0.000) | 89.079 (0.000) | 46.317 (0.236) |
| **Patient characteristics** |  |  |  |  |  |  |
| Age (centered) |  | .0554 (0.000) | .055 (0.000) | .055 (0.000) | .055 (0.000) | .055 (0.000) |
| Gender (ref=Man) |  | .516 (0.000) | .517 (0.000) | .517 (0.000) | .517 (0.000) | .517 (0.000) |
| Perceived health (ref=good)  - Poor  - Fair  - Very good |  | .378 (0.000)  .077 (0.515)  -.553 (0.000) | .378 (0.047)  .077 (0.518)  -.552 (0.000) | .377 (0.048)  .077 (0.518)  -.551 (0.000) | .377 (0.048)  .077 (0.518)  -.552 (0.000) | .378 (0.047)  .077 (0.519)  -.552 (0.000) |
| Chronic disease(s) |  | 1.485 (0.000) | 1.487 (0.000) | 1.486 (0.000) | 1.487 (0.000) | 1.486 (0.000) |
| Ethnicity(ref=non-immigrant)  - Second generation  - First generation |  | -.522 (0.031)  -.628 (0.001) | -.518 (0.032)  -.626 (0.001) | -.517 (0.033)  -.626 (0.001) | -.517 (0.032)  -.626 (0.001) | -.519 (0.032)  -.627 (0.001) |
| Education (ref=in between)  - lowest qualifications  - Highest qualifications |  | -.029 (0.819)  -.183 (0.128) | -.030 (0.814)  -.180 (0.133) | -.030 (0.816)  -.180 (0.135) | -.030 (0.814)  -.180 (0.133) | -.031 (0.810)  -.180 (0.134) |
| Hh. income (ref=average)  - below average  - above average |  | -.304 (0.008)  .368 (0.008) | -.300 (0.009)  .369 (0.017) | -.299 (0.009)  .369 (0.017) | -.300 (0.009)  .369 (0.017) | -.301 (0.009)  -.301 (0.009) |
| **FP characteristics** |  |  |  |  |  |  |
| Gender (ref=Man) |  | .258 (0.299) | .404 (0.108) | .403 (0.109) | .403 (0.109) | .413 (0.100) |
| Age (centered) |  | .002 (0.135) | .002 (0.158) | .002 (0.157) | .002 (0.159) | .002 (0.153) |
| **Workload** |  |  |  |  |  |  |
| Working hours |  |  | .052 (0.000) | -.047 (0.575) |  | .052 (0.000) |
| Consultation duration |  |  | .015 (0.502) | .016 (0.483) |  | .611 (0.156) |
| Job satisfaction |  |  | 1.443 (0.001) | -.111 (0.935) |  | 1.444342 (0.001) |
| **Deviations** |  |  |  |  |  |  |
| Hours deviation from national average |  |  |  |  | .052 (0.000) |  |
| Minutes deviation from national average |  |  |  |  | .015 (0.496) |  |
| Satisfaction deviation from national average |  |  |  |  | 1.452004 (0.000) |  |
| **Interactions** |  |  |  |  |  |  |
| Wrk hrs X satisfaction |  |  |  | .039 (0.230) |  |  |
| Values continuity (main effect) |  |  |  |  |  | 11.442 (0.345) |
| Continuity X minutes |  |  |  |  |  | .193 (0.145) |
| **Variance** |  |  |  |  |  |  |
| Country level (s.e.) | 103.682 (25.750) | 102.643 (25.492) | 102.760 (25.523) | 102.663 (25.499) | 102.478 (25.451) | 98.961 (24.597) |
| Practice level (s.e.) | 73.570 (1.507) | 71.929 (1.488) | 71.558 (1.481) | 71.539 (1.481) | 71.558 (1.481) | 71.529 (1.481) |
| Patient level (s.e.) | 105.037 (.661) | 104.148 (.675) | 104.147 (.675) | 104.147 (.675) | 104.147 (.675) | 104.147 (.675) |
| **ICCs** |  |  |  |  |  |  |
| ICC country | 36.7% | 36.8% | 36.9% | 36.9% | 36.8% | 36.0% |
| ICC GPs | 26.1% | 25.8% | 25.7% | 25.7% | 25.7% | 26.0% |

Appendix 3 (continued)

| N_i_=33  N_j_= 6,731  N_k_= 58,680 | Model 7 | Model 8 | Model 9 | Model 10 | Model 11 |
| --- | --- | --- | --- | --- | --- |
| Cons | 31.422 (0.431) | 34.793 (0.80) | 78.822 (0.000) | 79.592 (0.000) | 76.231 (0.000) |
| **Patient characteristics** |  |  |  |  |  |
| Age (centered) | .055 (0.000) | .055 (0.000) | .058 (0.000) | .058 (0.000) | .058 (0.000) |
| Gender (ref=Man) | .517 (0.000) | .517 (0.000) | .537 (0.000) | .537 (0.000) | .536 (0.000) |
| Perceived health (ref=good)  - Poor  - Fair  - Very good | .378 (0.047)  .077 (0.518)  -.552 (0.000) | .378 (0.047)  .077 (0.516)  -.552 (0.000) | .365 (0.072)  .065 (0.612)  -.669 (0.000) | .363 (0.073)  .065 (0.612)  -.669 (0.000) | .365 (0.071)  .066 (0.606)  -.669 (0.000) |
| Chronic disease(s) | 1.487 (0.000) | 1.487 (0.000) | 1.592 (0.000) | 1.594 (0.000) | 1.592 (0.000) |
| Ethnicity(ref=non-immigrant)  - Second generation  - First generation | -.519 (0.032)  -.627 (0.001) | -.518 (0.032)  -.627 (0.001) | -.714 (0.011)  -.701 (0.001) | -.711 (0.011)  -.698 (0.001) | -.711 (0.011)  -.702 (0.001) |
| Education (ref=in between)  - lowest qualifications  - Highest qualifications | -.030 (0.814)  -.180 (0.133) | -.030 (0.815)  -.180 (0.133) | -.084 (0.533)  -.245 (0.061) | -.084 (0.529)  -.245 (0.061) | -.084 (0.533)  -.245 (0.061) |
| Hh. income (ref=average)  - below average  - above average | -.300 (0.009)  .369 (0.017) | -.300 (0.009)  .369 (0.017) | -.324 (0.008)  .424 (0.014) | -.322 (0.008)  .425 (0.014) | -.323 (0.008)  .426 (0.014) |
| **FP characteristics** |  |  |  |  |  |
| Gender (ref=Man) | .393 (0.119) | .405 (0.107) | .371 (0.175) | .381 (0.164) | .364 (0.183) |
| Age (centered) | .002 (0.151) | .002 (0.160) | .003 (0.101) | .003 (0.103) | .003 (0.117) |
| **Workload** |  |  |  |  |  |
| Working hours | .231 (0.366) | .052 (0.000) | .060 (0.000) | .063 (0.003) | .060 (0.000) |
| Consultation duration | .0146 (0.513) | .015 (0.505) | .067 (0.087) | .013 (0.591) | .014 (0.579) |
| Job satisfaction | 1.426 (0.001) | 2.886 (0.725) | 1.590 (0.000) | 1.573 (0.001) | 2.926 (0.000) |
| **Interactions** |  |  |  |  |  |
| Values continuity (main effect) | 16.060 (0.194) | 15.010 (0.265) |  |  |  |
| values X hours | -.055 (0.483) |  |  |  |  |
| Values x satisfaction |  | -.448 (0.859) |  |  |  |
| List system (main effect) |  |  | 5.343 (0.163) | 4.283 (0.273) | 8.916 (0.048) |
| List X minutes |  |  | -.088 (0.077) |  |  |
| List X hours |  |  |  | -.007 (0.791) |  |
| List X satisfaction |  |  |  |  | -1.930 (0.050) |
| **Variance** |  |  |  |  |  |
| Country level (s.e.) | 98.776 (4.551) | 98.622 (24.513) | 101.036 (25.490) | 100.513 (25.359) | 100.849 (25.442) |
| Practice level (s.e.) | 71.552 (1.481) | 71.558 (1.481) | 76.885 (1.658) | 76.937 (1.659) | 76.875 (1.658) |
| Patient level (s.e.) | 104.147 (.675) | 104.147 (.675) | 111.339 (.752) | 111.338 (.752) | 111.339 (.752) |
| **ICCs** |  |  |  |  |  |
| ICC country | 36.0% | 36.0% | 35.0% | 34.8% | 34.9% |
| ICC GPs | 26.1% | 26.1% | 26.6% | 26.6% | 26.6% |

Appendix 4: Results linear multilevel analyses patient experiences with ***access*** (regression coefficients with p-values)

| N_i_=33  N_j_= 6,512  N_k_= 57,218 | Model 1 | Model 2 | Model 3 | Model 4 | Model 5 | Model 6 |
| --- | --- | --- | --- | --- | --- | --- |
| Cons | 85.024 (0.000) | 85.291 (0.000) | 81.704 (0.000) | 79.540 (0.000) | 85.253 (0.000) | 103.215 (0.000) |
| **Patient characteristics** |  |  |  |  |  |  |
| Age (centered) |  | .007 (0.000) | .007 (0.000) | .007 (0.000) | .007 (0.000) | .007 (0.000) |
| Gender (ref=Man) |  | .052 (0.004) | .052(0.003) | .052 (0.003) | .052 (0.003) | .052 (0.003) |
| Perceived health (ref=good)  - Poor  - Fair  - Very good |  | -.314 (0.000)  -.114 (0.000)  .088 (0.001) | -.314(0.000)  -.114(0.000)  .088(0.001) | -.314(0.000)  -.114(0.000)  .088(0.001) | -.314(0.000)  -.114(0.000)  .088(0.001) | -.314(0.000)  -.114(0.000)  .088(0.001) |
| Chronic disease(s) |  | .032 (0.120) | .032 (0.119) | .032 (0.119) | .032 (0.119) | .032 (0.119) |
| Ethnicity(ref=non-immigrant)  - Second generation  - First generation |  | -.132 (0.003)  -.391 (0.000) | -.132 (0.003)  -.391 (0.000) | -.132 (0.003)  -.391 (0.000) | -.132 (0.003)  -.391 (0.000) | -.132 (0.003)  -.391 (0.000) |
| Education (ref=in between)  - lowest qualifications  - Highest qualifications |  | -.099 (0.000)  .040 (0.071) | -.099 (0.000)  .040(0.071) | -.099 (0.000)  .040(0.071) | -.099 (0.000)  .040(0.071) | -.099 (0.000)  .040(0.071) |
| Hh. income (ref=average)  - below average  - above average |  | -.198 (0.000)  -.016 (0.574) | -.198 (0.000)  -.016 (0.574) | -.198 (0.000)  -.016(0.574) | -.198 (0.000)  -.016(0.574) | -.198 (0.000)  -.016(0.574) |
| **FP characteristics** |  |  |  |  |  |  |
| Gender (ref=Man) |  | -.276 (0.138) | -.207 (0.271) | -.206 (0.273) | -.016 (0.574) | -.207 (0.271) |
| Age (centered) |  | -.001 (0.384) | -.001 (0.359) | -.001 (0.357) | -.001 (0.361) | -.001 (0.362) |
| **Workload** |  |  |  |  |  |  |
| Working hours |  |  | .017 (0.062) | .072 (0.251) |  | .017 (0.062) |
| Consultation duration |  |  | -.001 (0.653) | -.008 (0.636) |  | -.198 (0.524) |
| Job satisfaction |  |  | 1.1934 (0.000) | 2.054 (0.044) |  | 1.195 (0.000) |
| **Deviations** |  |  |  |  |  |  |
| Hours deviation from national average |  |  |  |  | .016 (0.076) |  |
| Minutes deviation from national average |  |  |  |  | -.008 (0.633) |  |
| Satisfaction deviation from national average |  |  |  |  | 1.167 (0.000) |  |
| **Interactions** |  |  |  |  |  |  |
| Wrk hrs X satisfaction |  |  |  | -.022 (0.376) |  |  |
| Values access (main effect) |  |  |  |  |  | -6.859 (0.224) |
| Continuity X minutes |  |  |  |  |  | .060 (0.539) |
| **Variance** |  |  |  |  |  |  |
| Country level (s.e.) | 35.798 (8.910) | 35.641 (8.872) | 34.631 (8.629) | 34.614 (8.625) | 35.645 (8.873) | 33.603 (8.380) |
| Practice level (s.e.) | 47.641 (.844) | 47.400 (.841) | 47.280 (.839) | 47.275 (.839) | 47.280 (.839) | 47.276 (.839) |
| Patient level (s.e.) | 3.420 (.021) | 3.395 (.022) | 3.395 (.022) | 3.395 (.022) | 3.395 (.022) | 3.395 (.022) |
| **ICCs** |  |  |  |  |  |  |
| ICC country | 41.2% | 41.2% | 40.6% | 40.6% | 41.3% | 39.9% |
| ICC GPs | 54.8% | 54.8% | 55.4% | 55.4% | 54.8% | 56.1% |

Appendix 4 (continued)

| N_i_=33  N_j_= 6,731  N_k_= 58,680 | Model 7 | Model 8 | Model 9 | Model 10 | Model 11 |
| --- | --- | --- | --- | --- | --- |
| Cons | 108.541 (0.000) | 121.500 (0.000) | 82.794 (0.000) | 83.006 (0.000) | 83.846 (0.000) |
| **Patient characteristics** |  |  |  |  |  |
| Age (centered) | .007 (0.000) | .007 (0.000) | .006 (0.000) | .006 (0.000) | .006 (0.000) |
| Gender (ref=Man) | .052 (0.003) | .052 (0.003) | .056 (0.003) | .056 (0.003) | .056 (0.003) |
| Perceived health (ref=good)  - Poor  - Fair  - Very good | -.314 (0.000)  -.114 (0.000)  .088 (0.001) | -.314 (0.000)  -.114 (0.000)  .088 (0.001) | -.308 (0.000)  -.112 (0.000)  .086 (0.004) | -.308 (0.000)  -.112 (0.000)  .086 (0.004) | -.308 (0.000)  -.112 (0.000)  .086 (0.004) |
| Chronic disease(s) | .032 (0.119) | .032 (0.119) | .0278 (0.210) | .028 (0.2100 | .028 (0.209) |
| Ethnicity(ref=non-immigrant)  - Second generation  - First generation | -.133 (0.003)  -.391 (0.000) | -.132 (0.003)  -.391 (0.000) | -.164001)  -.411 (0.000) | -.164 (0.001)  -.411 (0.000) | -.164 (0.001)  -.411 (0.000) |
| Education (ref=in between)  - lowest qualifications  - Highest qualifications | -.099 (0.000)  .040 (0.071) | -.099 (0.000)  .040(0.071) | -.091 (0.000)  .045 (0.056) | -.091 (0.000)  .045 (0.056) | -.091 (0.000)  .045 (0.056) |
| Hh. income (ref=average)  - below average  - above average | -.198 (0.000)  -.016 (0.574) | -.198 (0.000)  -.016 (0.579) | -.209 (0.000)  -.017 (0.592) | -.209 (0.000)  -.017 (0.592) | -.209 (0.000)  -.017 (0.592) |
| **FP characteristics** |  |  |  |  |  |
| Gender (ref=Man) | -.204 (0.280) | -.209 (0.267) | -.236 (0.245) | -.239 (0.242) | -.233 (0.252) |
| Age (centered) | -.0001 (0.366) | -.001 (0.349) | -.002 (0.147) | -.002 (0.145) | -.002 (0.152) |
| **Workload** |  |  |  |  |  |
| Working hours | -.190 (0.249) | .0167 (0.064) | .014 (0.148) | .012 (0.444) | .014 (0.158) |
| Consultation duration | -.007 (0.666) | -.008 (0.650) | .006 (0.829) | .000 (0.998) | .000 (0.999) |
| Job satisfaction | 1.194 (0.000) | -7.125 (0.188) | 1.214 (0.000) | 1.212 (0.000) | .852 (0.165) |
| **Interactions** |  |  |  |  |  |
| Values access (main effect) | -8.607 (0.140) | -12.733 (0.070) |  |  |  |
| values X hours | .067 (0.208) |  |  |  |  |
| Values x satisfaction |  | 2.670 (0.124) |  |  |  |
| List system (main effect) |  |  | -1.915(0.398) | -2.224 (0.342) | -3.3764 (0.241) |
| List X minutes |  |  | -.010 (0.788) |  |  |
| List X hours |  |  |  | .004 (0.848) |  |
| List X satisfaction |  |  |  |  | .513 (0.484) |
| **Variance** |  |  |  |  |  |
| Country level (s.e.) | 33.381 (8.318) | 34.020 (8.486) | 34.262 (8.686) | 34.233 (8.677) | 34.068 (8.638) |
| Practice level (s.e.) | 47.269 (.839) | 47.258 (.839) | 49.951 (.924) | 49.9516 (.924) | 49.949 (.924) |
| Patient level (s.e.) | 3.394 (.022) | 3.3945 (.022) | 3.557 (.024) | 3.557 (.024) | 3.557 (.024) |
| **ICCs** |  |  |  |  |  |
| ICC country | 39.7% | 40.2% | 39.0% | 39.0% | 38.9% |
| ICC GPs | 56.2% | 55.8% | 56.9% | 56.9% | 57.0% |

Appendix 5: Results linear multilevel analyses patient experiences with ***comprehensiveness*** (regression coefficients with p-values)

| N_i_=33  N_j_= 6,512  N_k_= 56,808 | Model 1 | Model 2 | Model 3 | Model 4 | Model 5 | Model 6 |
| --- | --- | --- | --- | --- | --- | --- |
| Cons | 68.390 (0.000) | 67.738 (0.000) | 60.216 (0.000) | 61.567 (0.000) | 67.729 (0.000) | 34.731 (0.123) |
| **Patient characteristics** |  |  |  |  |  |  |
| Age (centered) |  | .005 (0.000) | .005 (0.000) | .005 (0.000) | .005 (0.000) | .005 (0.000) |
| Gender (ref=Man) |  | .039 (0.089) | .039 (0.089) | .039 (0.089) | .039 (0.089) | .039 (0.089) |
| Perceived health (ref=good)  - Poor  - Fair  - Very good |  | .122 (0.007)  .023 (0.414)  -.033 (0.350) | .121 (0.007)  .023(0.416)  -.033 (0.350) | .121 (0.007)  .023(0.416)  -.033 (0.350) | .121 (0.007)  .023(0.416)  -.033 (0.350) | .121 (0.007)  .023(0.415)  -.033 (0.349) |
| Chronic disease(s) |  | .224 (0.000). | .224 (0.000) | .224 (0.000) | .224 (0.000) | .224 (0.000) |
| Ethnicity(ref=non-immigrant)  - Second generation  - First generation |  | -.162 (0.005)  -.008 (0.858) | -.162 (0.005)  -.009 (0.855) | -.162 (0.005)  -.009 (0.855) | -.162 (0.005)  -.009 (0.855) | -.162 (0.005)  -.009 (0.854) |
| Education (ref=in between)  - lowest qualifications  - Highest qualifications |  | -.003 (0.926)  -.064 (0.025) | -.003 (0.929)  -.064 (0.025) | -.003 (0.929)  -.064 (0.025) | -.003 (0.929)  -.064 (0.025) | -.003 (0.928)  -.064 (0.025) |
| Hh. income (ref=average)  - below average  - above average |  | .010 (0.710)  .005 (0.897) | .010 (0.704)  .005 (0.895) | .010 (0.703)  .005 (0.895) | .010 (0.704)  .005 (0.895) | .010 (0.703)  .005 (0.896) |
| **GP characteristics** |  |  |  |  |  |  |
| Gender (ref=Man) |  | 1.035 (0.000) | 1.051 (0.000) | 1.051 (0.000) | 1.048 (0.000) | 1.058 (0.000) |
| Age (centered) |  | .002 (0.166) | .002 (0.235) | .002 (0.235) | .002 (0.235) | .002 (0.227) |
| **Workload** |  |  |  |  |  |  |
| Working hours |  |  | .038 (0.007) | .004 (0.968) |  | .038 (0.007) |
| Consultation duration |  |  | .108 (0.000) | .109 (0.000) |  | -.167 (0.623) |
| Job satisfaction |  |  | 1.763 (0.000) | 1.226 (0.441) |  | 1.754 (0.000) |
| **Deviations** |  |  |  |  |  |  |
| Hours deviation from national average |  |  |  |  | .037 (0.009) |  |
| Minutes deviation from national average |  |  |  |  | .108 (0.000) |  |
| Satisfaction deviation from national average |  |  |  |  | 1.756 (0.000) |  |
| **Interactions** |  |  |  |  |  |  |
| Wrk hrs X satisfaction |  |  |  | .014 (0.723) |  |  |
| Values compr. (main effect) |  |  |  |  |  | 8.461 (0.255) |
| Continuity X minutes |  |  |  |  |  | .090 (0.416) |
| **Variance** |  |  |  |  |  |  |
| Country level (s.e.) | 90.303 (22.494) | 90.391 (22.515) | 88.420 (22.040) | 88.399 (22.035) | 90.381 (22.511) | 84.093 (20.985) |
| Practice level (s.e.) | 116.120 (2.052) | 115.850 (2.050) | 115.212 (2.039) | 115.210 (2.039) | 115.212 (2.039) | 115.198 (2.039) |
| Patient level (s.e.) | 5.705 (.036) | 5.688 (.037) | 5.688 (.037) | 5.688 (.037) | 5.688 (.037) | 5.688 (.037) |
| **ICCs** |  |  |  |  |  |  |
| ICC country | 42.6% | 42.7% | 42.2% | 42.2% | 42.8% | 41.0% |
| ICC GPs | 54.7% | 54.7% | 55.0% | 55.0% | 54.5% | 97.2% |

Appendix 5 (continued)

| N_i_=33  N_j_= 6,731  N_k_= 58,680 | Model 7 | Model 8 | Model 9 | Model 10 | Model 11 |
| --- | --- | --- | --- | --- | --- |
| Cons | 24.236 (0.291) | 14.538 (0.585) | 59.892 (0.000) | 61.320 (0.000) | 58.301 (0.000) |
| **Patient characteristics** |  |  |  |  |  |
| Age (centered) | .005 (0.000) | .005 (0.000) | .005 (0.000) | .005 (0.000) | .005 (0.000) |
| Gender (ref=Man) | .039 (0.089) | .039 (0.089) | .052 (0.033) | .052 (0.033) | .052 (0.033) |
| Perceived health (ref=good)  - Poor  - Fair  - Very good | .121 (0.007)  .023 (0.415)  -.033 (0.349) | .121 (0.007)  .023 (0.415)  -.033(0.349) | .129 (0.006)  .021 (0.486)  -.044 (0.243) | .129 (0.006)  .021 (0.487)  -.044 (0.243) | .129 (0.006)  .021 (0.486)  -.044 (0.243) |
| Chronic disease(s) | .224 (0.000) | .224(0.000) | .230 (0.000) | .230 (0.000) | .230 (0.000) |
| Ethnicity(ref=non-immigrant)  - Second generation  - First generation | -.162 (0.005)  -.009 (0.854) | -.162 (0.005)  -.009 (0.854) | -.171 (0.009)  .003 (0.947) | -.171(0.009)  .003 (0.947) | -.171(0.009)  .003 (0.947) |
| Education (ref=in between)  - lowest qualifications  - Highest qualifications | -.003 (0.929)  -.064 (0.025) | -.003 (0.929)  -.064 (0.025) | -.007 (0.834)  -.063 (0.040) | -.007 (0.833)  -.063 (0.040) | -.007 (0.834)  -.063 (0.040) |
| Hh. income (ref=average)  - below average  - above average | .010 (0.704)  .005 (0.896) | .010 (0.703)  .005 (0.895) | .010 (0.723)  -.002 (0.952) | .010 (0.722)  -.002 (0.953) | .010 (0.722)  -.002 (0.954) |
| **GP characteristics** |  |  |  |  |  |
| Gender (ref=Man) | 1.038 (0.000) | 1.055 (0.000) | 1.001 (0.002) | .996 (0.002) | .998 (0.002) |
| Age (centered) | .002 (0.219) | .002 (0.248) | .003 (0.079) | .003 (0.085) | .003 (0.089) |
| **Workload** |  |  |  |  |  |
| Working hours | .214 (0.209) | .037 (0.008) | .044 (0.005) | .035 (0.154) | .043 (0.006) |
| Consultation duration | .108 (0.000) | .108 (0.000) | .160 (0.000) | .100 (0.001) | .100 (0.001) |
| Job satisfaction | 1.731 (0.000) | 8.506 (0.172) | 1.917 (0.000) | 1.895 (0.000) | 2.920 (0.002) |
| **Interactions** |  |  |  |  |  |
| Values compr. (main effect) | 11.939 (0.115) | 15.108 (0.086) |  |  |  |
| values X hours | -.0582 (0.300) |  |  |  |  |
| Values x satisfaction |  | -2.219 (0.276) |  |  |  |
| List system (main effect) |  |  | -.746 (0.835) | -2.789 (0.448) | 1.440 (0.750) |
| List X minutes |  |  | -.100 (0.083) |  |  |
| List X hours |  |  |  | .012 (0.692) |  |
| List X satisfaction |  |  |  |  | -1.459 (0.202) |
| **Variance** |  |  |  |  |  |
| Country level (s.e.) | 84.125 (20.992) | 84.077 (20.979) | 85.338 (21.659) | 84.5973 (21.470) | 84.837 (21.530) |
| Practice level (s.e.) | 115.191 (2.038) | 115.189 (2.038) | 121.629 (2.244) | 121.694 (2.245) | 121.661 (2.244) |
| Patient level (s.e.) | 5.688 (.037) | 5.688 (.037) | 5.913 (.040) | 5.913 (.040) | 5.913 (.040) |
| **ICCs** |  |  |  |  |  |
| ICC country | 41.0% | 41.0% | 40.1% | 39.9% | 39.9% |
| ICC GPs | 56.2% | 56.2% | 57.1% | 57.3% | 57.3% |
